# Afferent and Efferent Connections of the Cortex-Amygdala Transition Zone in Mice

**DOI:** 10.3389/fnana.2016.00125

**Published:** 2016-12-23

**Authors:** Bernardita Cádiz-Moretti, María Abellán-Álvaro, Cecília Pardo-Bellver, Fernando Martínez-García, Enrique Lanuza

**Affiliations:** ^1^Laboratori de Neuroanatomia Funcional Comparada, Departament de Biologia Cel⋅lular i Biologia Funcional, Facultat de Ciències Biològiques, Universitat de ValènciaValència, Spain; ^2^Unitat Predepartamental de Medicina, Facultat de Ciències de la Salut, Universitat Jaume ICastelló de la Plana, Spain

**Keywords:** vomeronasal, olfactory, piriform cortex, amygdala, neural tracing

## Abstract

The transitional zone between the ventral part of the piriform cortex and the anterior cortical nucleus of the amygdala, named the cortex-amygdala transition zone (CxA), shows two differential features that allow its identification as a particular structure. First, it receives dense cholinergic and dopaminergic innervations as compared to the adjacent piriform cortex and amygdala, and second, it receives projections from the main and accessory olfactory bulbs. In this work we have studied the pattern of afferent and efferent projections of the CxA, which are mainly unknown, by using the retrograde tracer Fluorogold and the anterograde tracer biotinylated dextranamine. The results show that the CxA receives a relatively restricted set of intratelencephalic connections, originated mainly by the olfactory system and basal forebrain, with minor afferents from the amygdala. The only relevant extratelencephalic afference originates in the ventral tegmental area (VTA). The efferent projections of the CxA reciprocate the inputs from the piriform cortex and olfactory amygdala. In addition, the CxA projects densely to the basolateral amygdaloid nucleus and the olfactory tubercle. The extratelencephalic projections of the CxA are very scarce, and target mainly hypothalamic structures. The pattern of connections of the CxA suggests that it is indeed a transitional area between the piriform cortex and the cortical amygdala. Double labeling with choline acetyltransferase indicates that the afferent projection from the basal forebrain is the origin of its distinctive cholinergic innervation, and double labeling with dopamine transporter shows that the projection from the VTA is the source of dopaminergic innervation. These connectivity and neurochemical features, together with the fact that it receives vomeronasal in addition to olfactory information, suggest that the CxA may be involved in processing olfactory information endowed with relevant biological meaning, such as odors related to reproductive or defensive behaviors.

## Introduction

The ventralmost part of the piriform cortex adjacent to the rostral amygdala can be recognized as a different area in rodents, according to some particular cyto- and chemoarchitectural features. Since it is contiguous to the amygdaloid complex, it was named as cortex-amygdala transition zone (CxA) in the first edition of the rat brain atlas by Paxinos and Watson (1982), which is, to the best of our knowledge, the first time the CxA was recognized as such. The local histochemical detection of acetyl cholinesterase activity clearly showed that the CxA was different from the adjacent piriform cortex (Pir) and neighboring amygdaloid structures (Paxinos and Watson, 1982). The same neurochemical feature allows distinguishing the CxA in the mouse brain (Paxinos and Franklin, 2004; Martínez-García et al., 2012). In addition to the acetyl cholinesterase reactivity, the CxA also shows a denser dopaminergic innervation than its adjacent piriform and amygdalar formations, as revealed by the immunohistochemical detection of tyrosine hydroxylase (Martínez-García et al., 2012).

The rostrocaudal extent of the CxA is much more restricted than that of the Pir. In the mouse brain, it appears rostrally together with the anterior amygdaloid area, and extends caudally until the appearance of the posterolateral cortical amygdaloid nucleus (PLCo). Therefore, in most of its extent the CxA is interposed between the ventral Pir and the anterior cortical amygdaloid nucleus (ACo) (Paxinos and Franklin, 2004; Martínez-García et al., 2012). It is worth to note that the CxA does not correspond to the piriform-amygdalar area of the Allen Brain Atlas, which is more caudally located, at the levels of the PLCo. In addition, it should not be confused with the amygdalopiriform transition area (APir) (Paxinos and Franklin, 2004), located at the caudal edge of the Pir, which is interposed between the amygdala and the entorhinal cortex. Finally, the CxA does not correspond to the ventrorostral anterior Pir described by Ekstrand et al. (2001), which is located further rostrally.

From the hodological point of view, the CxA also differs from the adjacent Pir in the reception of a small projection from the AOB, both in rats (Pro-Sistiaga et al., 2007) and mice (Cádiz-Moretti et al., 2013).

Therefore, in contrast to the neighboring Pir, the CxA receives cholinergic and dopaminergic inputs, as well as a (minor) vomeronasal input by means of a direct projection from the accessory olfactory bulb (AOB). The convergence of vomeronasal information with cholinergic and dopaminergic signaling may suggest that the CxA is more involved in processing the odor information related with predators or possible mates (that is, with particular salience), rather than the processing of general olfactory information. Alternatively, given the known role of cholinergic (Wilson and Fadel, 2016) and dopaminergic (Lee et al., 2016) innervation of the amygdala in plasticity, it is possible that this particular neurochemical features are related to the occurrence of olfactory learning processes in the CxA. However, the lack of information about the rest of the connections of this structure dampens a better understanding about its possible roles. To our knowledge, with the exception of the projections from the olfactory bulbs, no studies are available describing the connections of the CxA. In their seminal studies about the organization and connectivity of the olfactory cortex, Price and collaborators (Haberly and Price, 1978a,b; Luskin and Price, 1983) described two systems of longitudinal connections: the layer Ib system, originated by the anterior olfactory nucleus, the Pir and the entorhinal cortex; and the layer II system, which arises from the dorsal peduncular cortex (DP), the ventral tenia tecta (VTT) and the periamygdaloid cortex. In case the CxA is more related to the Pir than to the amygdala, it would probably belong to the Ib system, whereas in case it is more related to the cortical amygdala, then it would probably belong to the layer II system. Since the CxA is not widely recognized by the scientific community working in the amygdala or in the olfactory system, the present work may contribute to clarify whether, according to its connections, it is really a transition area between the Pir and the cortical amygdala, or it should be considered simply part of the Pir, or part of the amygdala.

In summary, the aim of this work is to provide a comprehensive description of the afferent and efferent connections of the CxA, to help understanding the possible role of this structure in processing convergent vomeronasal and olfactory information, as well as in olfactory plasticity maybe related to the arousing properties of biologically relevant odor cues (such as those derived from predators or conspecifics).

## Materials and Methods

### Animals

For this study, we used 17 adult female mice (*Mus musculus*) from the CD1 strain (Janvier, Le Genest Saint-Isle, France), which were 8–27 weeks old and weighed 27.8–48.7 g at the beginning of the experiments. Animals were housed in cages with water and food available *ad libitum* with 12 h light: dark cycle at 22–24°C. The mice were treated according to the guidelines of the European Union Council Directive of June 3rd, 2010 (6106/1/10 REV1). All the experimental procedures were approved by the Committee of Ethics on Animal Experimentation of the University of Valencia.

### Surgery and Tracer Injections

During surgery, animals were kept under appropriate levels of anesthesia with isofluorane (2-2.5%) in oxygen (1-1.3 L/min) (MSS Isoflurane Vaporizer, Medical Supplies and Services, UK) delivered through a mouse anesthetic mask coupled to the stereotaxic apparatus. They also received an analgesic injection of butorphanol (5 mg/kg, Turbugesic, Pfizer, New York, NY, USA) subcutaneously. After fixing the mouse head in the stereotaxic frame (David Kopf, 963-A, Tujunga, CA, USA) and adjusting the position of the head so that bregma and lambda were disposed in the same plane, a small hole was drilled above the target zone.

To study the afferent and efferent projections of CxA, mice received iontophoretic injections of the fluorescent retrograde tracer Fluorogold (FG) [hydroxystilbamidine *bis* (methanesulfonate), Sigma–Aldrich, Cat # 39286] diluted at 2% in distilled water and of the anterograde tracer biotin-conjugated dextranamine (BDA, 10,000 MW, lysine fixable, Invitrogen, Carlsbad, CA, USA), diluted at 5% in phosphate buffer (PB, 0.01M, pH 8.0). To reduce the number of animals used in the study, in each animal we injected FG in one hemisphere and BDA in the other. We delivered the tracers from glass micropipettes (20–30 μm diameter tips) by means of positive current pulses (7on/7off) of 2–3 μA during 3–6 min, and the micropipette was left in place for 10 min after finishing the injection. To avoid diffusion of the tracer along the pipette track, we applied a mild continuous retention current (-0.1 μA) during the entrance and withdrawal of the micropipette. Injection coordinates relative to bregma (AP -0.09 to -0.2 mm, L -2.89 to -2.94 mm, DV -6.04 mm) were taken from the atlas of the mouse brain (Paxinos and Franklin, 2004).

After the injection, we closed the wound with Histoacryl (Braun, Tuttlinger, Germany). Animals rested on a thermal blanket to maintain their body temperature during the whole procedure. In addition, they received eye drops (Siccafluid, Thea S.A. Laboratories, Spain) to prevent eye ulceration.

### Histology

After 7–8 days of survival, animals received an overdose of sodium pentobarbital (intraperitoneal, 100 mg/kg, Eutanax, Laboratorios Normon S. A. Madrid, Spain) and were transcardially perfused with saline solution (0.9%) followed by 4% paraformaldehyde diluted in PB (0.1M, pH 7.6). Then, brains were carefully removed from the skulls, postfixed for 4 h in the same fixative and cryoprotected in 30% sucrose in PB (0.1M, pH 7.6) at 4°C until they sank. The olfactory bulbs were cut in 30 μm-thick sagittal sections, and frontal sections (40 μm) were obtained from the rest of the brain using a freezing microtome. In both cases, sections were collected in four parallel series.

The location of the FG injections was checked using fluorescence microscopy. In addition, we processed one of the series of each brain for the simultaneous (immuno)histochemical detection of BDA and FG in free-floating sections. For the detection of the BDA, endogenous peroxidase was inactivated with 1% H_2_O_2_ in Tris-buffered saline (TBS; 0.05 M, pH 7.6) for 15 min at room temperature and then sections were incubated for 90 min in ABC complex (Vectastain ABC kit, Vector Labs, PK-6100, Burlingame, CA, USA) diluted 1:50 in TBS-Tx (0.3% Triton X-100 in 0.05 M TBS, pH 7.6). After rinsing the tissue with TBS, peroxidase activity was developed with 0.025% diaminobenzidine in Tris buffer (TB; 0.05 M, pH 8.0) and 0.01% H_2_O_2_ and 0.1% nickel ammonium sulfate. The resulting reaction product was a black precipitate. Subsequently, the same tissue was processed for immunoperoxidase detection of FG. First, sections were incubated in a blocking solution of TBS-Tx containing 8% normal goat serum (NGS) and 4% bovine seroalbumin (BSA) for 2 h at room temperature. After that, the sections were sequentially incubated in: (a) rabbit anti-FG (Millipore, Cat # AB153) diluted 1:3000 in TBS-Tx with 4% NGS and 2% BSA overnight at 4°C; (b) biotinylated goat anti-rabbit IgG (Vector, Cat # BA-1000) diluted 1:200 in TBS-Tx with 4% NGS for 2 h at room temperature; and (c) ABC Elite diluted 1:50 in TBS-Tx for 2 h at room temperature. Finally, the resulting peroxidase labeling was developed with 0.025% DAB in TB (0.1M, pH 8.0) with 0.01% H_2_O_2_. The resulting precipitate was a brown product, easily distinguishable from the black precipitate resulting from the histochemical detection of BDA. For some of the animals, an additional series was processed only for the detection of BDA and counterstained with Nissl. The omission of the primary antibody or performing the immunohistochemical procedure in animals with no FG injection yielded no labeling.

Sections were mounted onto gelatinized slides, dehydrated in alcohols, cleared with xylene and cover-slipped with Entellan (Merck Millipore).

In addition, some series of the cases with restricted injections of FG were processed using immunofluorescence techniques to study the co-localization of FG, dopamine transporter (DAT) and choline acetyltransferase (ChAT). Firstly, sodium borohydride (diluted 1% in TBS) was used as an aldehyde-blocking reagent for 30 min at room temperature. After that, sections were incubated in a blocking solution containing 4% normal horse serum and 0.4% Triton X-100 in TBS for 2 h at room temperature, followed by a mixture of the three primary antibodies (in the same blocking solution) at the following concentrations: goat anti-ChAT (Millipore; Cat # AB144P) diluted 1:200, rabbit anti-FG (Millipore; Cat # AB153) diluted 1:1000 and rat anti-DAT (Chemicon International; Cat # MAB369) diluted 1:1000, for 72 h at 4°C. After rinsing, the sections were incubated with the following fluorescent secondary antibodies, diluted 1:200 in blocking solution: Alexa 488 donkey anti-rat (Jackson Immunoresearch; Cat # 712-545-153) and Rhodamine (TRITC) donkey anti-goat (Jackson Immunoresearch; Cat # 705-025-147). Finally, to avoid binding with the anti-goat antibody, the incubation with Cascade Blue-conjugated goat anti-rabbit (LifeTechnologies; Cat # C2764) was done alone, as a final step, with a previous incubation in a blocking solution of 4% normal goat serum in 0.4% TBS-Tx for 1 h at room temperature. Sections were mounted onto gelatinized slides and cover-slipped with fluorescence mounting medium (Dako).

We also used series of Nissl-stained brain sections as well as series processed for the immunohistochemical detection of DAT and ChAT, available at the laboratory. The DAT (Martínez-Hernández et al., 2012) and ChAT immunohistochemistry were performed as described above for the FG, using the primary antibody rat anti-DAT (Chemicon International, Cat # MAB369) diluted 1:2000 and goat anti-ChAT (Millipore; Cat # AB144P) diluted 1:500 respectively, the biotinylated secondary antibodies rabbit anti-rat and horse anti-goat (diluted 1:200, Vector Labs.) and ABC Elite. The resulting peroxidase reactivity was visualized with 0.04% DAB in TB, with 0.01% H_2_O_2_ as substrate. In the case of the DAT immunohistochemistry, nickel ammonium sulfate (0.4%) was used as enhancer.

### Image Acquisition and Processing

We observed the sections using an Olympus CX41RF-5 microscope and photographed them using a digital Olympus XC50 camera. Fluorescent images of the FG injection sites were captured with a Leitz DMRB microscope with epifluorescence (Leica EL-6000) equipped with a specific filter for FG (Leica, A) and a digital Leica DFC 300 FX camera. The triple inmunofluorescent preparations were observed using a Leica TCS SP8 inverted confocal microscope and the images were obtained with the Leica Application Suite X (LASX, Leica Microsystems). Using Adobe Photoshop CS6 (Adobe Systems, San Jose, CA, USA) pictures were flattened by subtracting background illumination and brightness and contrast were optimized. No further changes were performed.

The mapping of brain regions labeled after the injections was carried out using one of the restricted injections as a model (case 1324 for afferent projections, and case 1331 for efferent projections). To do so, we imported selected photographs into Adobe Illustrator CS6 (Adobe Systems) and drew the nuclear boundaries using the atlas by Paxinos and Franklin (2004) as a reference. Finally, labeled fibers and somata were charted according to the projection or the population density, respectively. The labeling density was considered as very dense, dense, moderate, scarce, and very scarce (see **Table 1**). As a reference, we considered very dense the retrograde labeling in the mitral cell layer of the olfactory bulb, and very scarce the presence of only 2–5 labeled cell bodies. In the case of anterograde labeling, we considered very dense the labeling observed in the posterior division of the basolateral nucleus, and very scarce the presence of only 2–5 labeled fibers.

**Table 1:** Qualitative rating of the density of the retrograde and anterograde labeling resulting after tracer injections in CxA.

		Retrograde	Anterograde
**Olfactory system**			
Accessory olfactory bulb	MiA	+	↓ +
	GrA	0	↓ +
Main olfactory bulb	GrO	0	+
	Mi	++++	0
Olfactory cortex	DTT/VTT	+/0	+
	AOL	0	+
	AOV	0	+
	AOM	0	+
	AOP	↓+	+
	rostral Pir	++	+
	caudal Pir	+	++
	DEn/VEn	++/+	++
**Amygdala and BST**			
Vomeronasal amygdala	AAV/AAD	+/+	+++
	Baot	++	+++
	MeA	+	+++
	MeAV/MeAD	+/+	++++
	MePV	↓+	+
	MePD	0	+
	PMCo	↓+	++
Olfactory amygdala	CxA	Injection	Injection
	LOT	+	++++
	ACo	++	+++
	PLCo	+	+++
	APir	0	+++
Basolateral complex	BLA	0	+++/+
	BLP	0	++++
	BLV	0	++++
	BMA	↓+	+++
	BMP	0	+++
	La	0	↓ +
Amygdalohippocampal transition area	AHi	0	+
Central	CeC	0	↓ +
	CeL	0	↓ +
	CeM	↓+	++
	Astr	0	+
	I	0	+++
	IM	0	++
BST	BSTLD	0	↓ +
	BSTLV	0	↓ +
	BSTLP	0	↓ +
	BSTMA	0	↓ +
	BSTMV	0	↓ +
	BSTMPM	0	↓ +
	BSTMPI	0	↓ +
	BSTMPL	0	↓ +
	BSTIA	0	+++
**Cortex**			
Cortex	AID	0	↓ +
	Al	0	↓ +
	AIP	↓+	↓ +
	Cl	0	↓ +
	MO	0	↓ +
	LO	0	↓ +
	IL	0	↓ +
	Ect	0	↓ +
	DP	0	↓ +
	PRh	0	+++
	LEnt	+	++
**Septum/Basal forebrain**			
Lateral septal complex	LSI	0	↓ +
	SHi	↓+	0
	SHy	0	↓ +
Medial septum/ Diagonal band	HDB/MCPO	++	++
	VDB	+	↓ +
Striato-pallidum	VP	+	+
	ICj	0	++
	Acb	0	++
	Tu	0	++
	SL	+	+
	SI	↓+	++
	IPAC	↓+	↓ +
**Preoptic area**			
Preoptic	MPA	0	↓ +
	LPO	0	↓+
**Hypothalamus**			
Anterior region	AHA/AHP	0	↓+/↓+
	LA	0	↓+
Tuberal region	RCh	0	↓+
	VMH	0	↓+
	DM	0	↓+
	MCLH	0	+
	LH	0	+
	PH	0	↓+
	TC	0	↓+
	PSTh	0	↓ +
Mamillary region	PMD/PMV	0	↓ +
	MM/ML	0	↓ +
	SuM	0	+
**Prethalamus and thalamus**			
	ZI	0	↓+
	PVA/PV	0	↓+
	PVP	0	↓+


**Midbrain and brainstem**			


	VTA	++	0
	RLi	↓+	0


	DR	+	0
	IP	0	↓+


	MPB	0	↓+

*For abbreviations, see list. ++++ very dense; +++ dense; ++ moderate; + scarce; ↓+ very scarce; 0 not found*.

## Results

For the description of the results, we follow the cytoarchitecture and nomenclature by Paxinos and Franklin (2004). Following Kevetter and Winans (1981b), the term “olfactory amygdala” is used for the amygdaloid structures that are direct targets of the MOB, and the term “vomeronasal amygdala” refers to the amygdaloid structures that are direct targets of the AOB (Kevetter and Winans, 1981a). The term “chemosensory amygdala” includes both the olfactory and the vomeronasal amygdala (Gutierrez-Castellanos et al., 2010).

### Cyto- and Chemoarchitecture of the CxA

**FIGURE 1:**
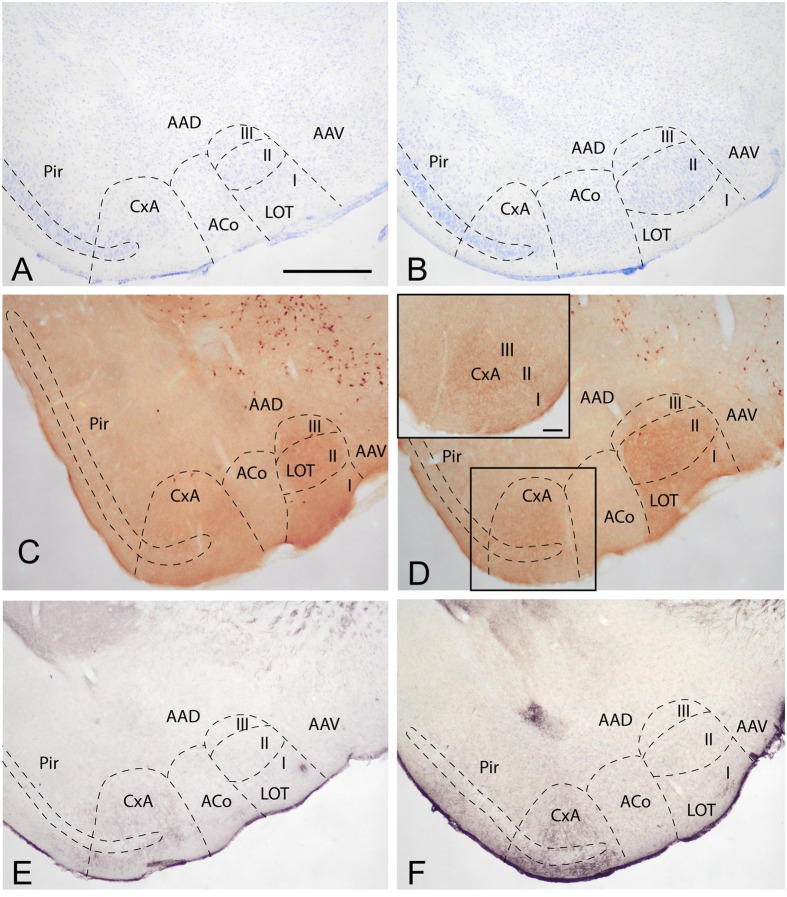
**Cyto- and chemoarchitecture of the CxA in the mouse brain**. Coronal Nissl-stained sections of the mouse telencephalon through anterior and posterior levels of the CxA **(A,B)**. Similar rostrocaudal levels processed for the immunodetecion of choline acetyltransferase (ChAT) **(C,D)** and dopamine transporter (DAT) **(E,F)**. Note the differences in ChAT and DAT reactivity between the CxA and the anterior cortical amygdala **(C,F)** and the differences in their laminar organization **(A,B)**. Scale bar in **A**: 0.5 mm.

To determine the boundaries, extent and cytoarchitectonic organization of the CxA, we used series of brain section stained with Nissl, or processed for the immunodetection of ChAT or DAT (**Figure 1**).

In Nissl-stained material, the CxA looks like a ventral extension of the piriform cortex (Pir), with the typical trilaminar organization of paleocortical structures (**Figures 1A,B**). However, in contrast to the Pir, layer III of the CxA shows a relatively reduced size with a lower density of large pyramidal cells typical of this stratum. ChAT immunohistochemistry reveals another differential property of CxA relative to the adjoining Pir, namely the presence of a plexus of positive fibers in layer III and, to a lesser extent, also in layers I and II. In addition, ChAT immunoreactivity reveals a clear-cut boundary of the CxA with the ACo (**Figures 1C,D**). Similar to the distribution of ChAT immunoreactivity, the DAT immunoreactivity in the cortical amygdala is concentrated in the CxA, being virtually absent in the ACo and relatively light in the adjacent Pir (**Figures 1E,F**).

According to these cyto- and chemoarchitectonic criteria, the rostral edge of the CxA lies between the anterior amygdala and the Pir (slightly rostral to the level shown in **Figure 1A**), with its deep aspect limiting with the VEn and the dorsal anterior amygdaloid area. More caudally, the CxA lies between the ACo and the Pir, and its deep aspect lacks a clear limit with the extension of layer III of the Pir. Even more caudally, the intensity of the ChAT and DAT immunoreactivity decreases gradually, so that, in its caudal edge, the CxA is not clearly distinguishable from the adjacent Pir. At the level of the PLCo the CxA is no longer present. Therefore, for most of its rostrocaudal extent, the CxA is interposed between the ACo (medially) and the rostral aspect of the posterior Pir (see Martínez-García et al., 2012). In this regard, it is worth to note that the Pir is divided into a rostral part (or prepiriform area, see Haberly and Price, 1978a,b) characterized by the presence of the lateral olfactory tract and a clear limit with the olfactory tubercle, and a caudal part where the lateral olfactory tract is not observable and there is no clear-cut boundary with the CxA. Therefore, the CxA appears just at the same anteroposterior level than the posterior Pir.

### Retrograde Labeling after FG Injections into the CxA

**FIGURE 2:**
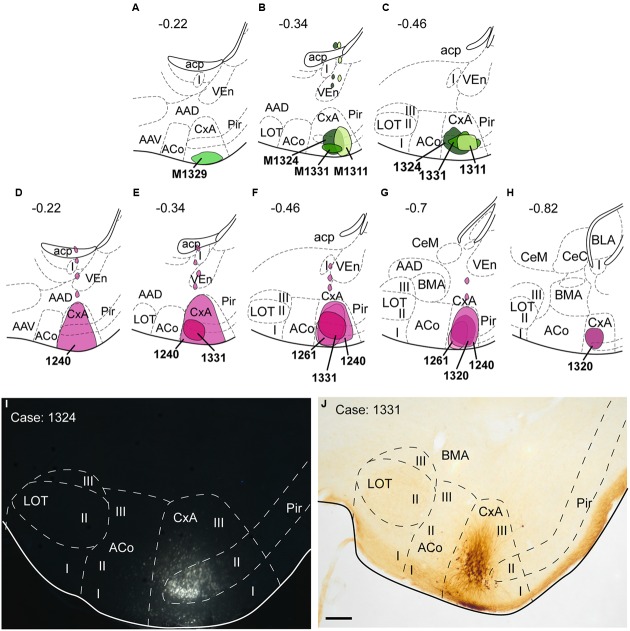
**Schematic drawings representing the extent of the injection sites of FG and BDA restricted to the CxA**. The injections of FG (retrograde tracer) are represented in green in **(A–C)**; the injections of BDA (anterograde tracer) are shown in purple in **(D–H)**. Single injections are identified with the animal code. **(I,J)**: Photomicrographs through the amygdala showing representative injections sites of FG (**I**, fluorescence microscopy) and BDA **(J)**. Scale bar in **(J)**, valid for **(I)** = 200 μm.

**FIGURE 3:**
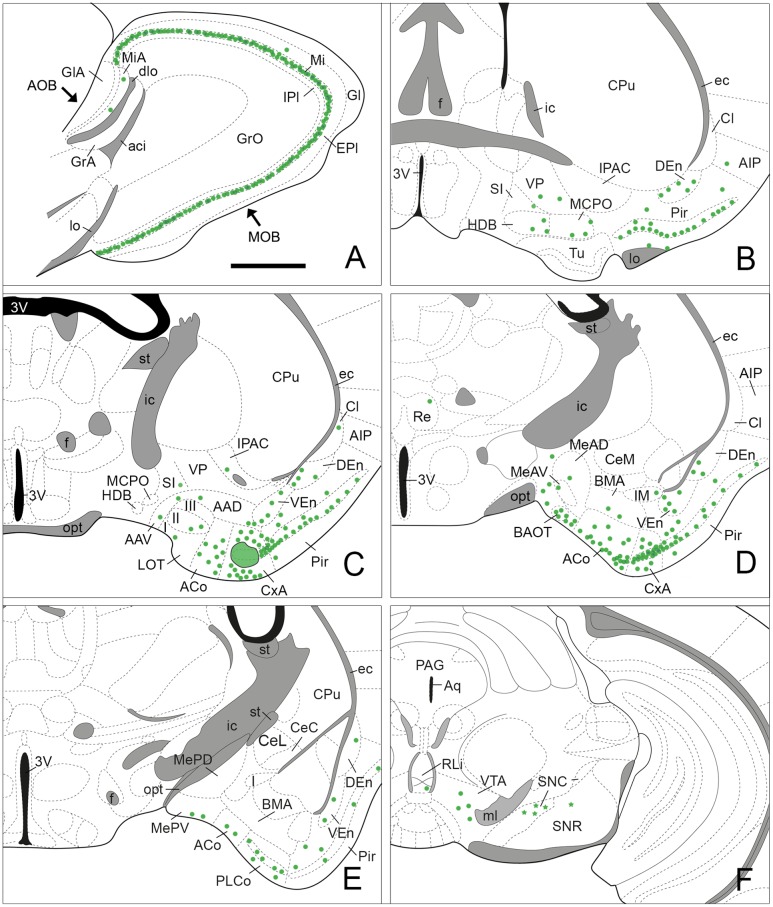
**Semi-schematic drawings of parasagittal** **(A)** and frontal **(B–F)** sections through the mouse brain showing the distribution of retrogradely labeled somata following a FG tracer injection in the CxA. The injection site is depicted in **(C)**. The semi-schematic drawings are based on the case 1324, which presented the largest restricted injection located in layer II. **(B)** is rostral, **(F)** is caudal. For abbreviations, see list. Scale bar in **(A)**: 800 μm. Scale in **(B)** (valid for **C–F**): 1.2 mm.

Four restricted injections were obtained in the CxA (**Figures 2A–C,I**), two of which involved the most superficial part of its layer I and probably the lateral olfactory tract (*lo*) (1311 and 1329). The other two (1324 and 1331) were centered in the cell-dense layer II. In three of these injections (1329, 1324, 1311), a small deposit of the tracer could be observed along the micropipette track in the ventral endopiriform nucleus (VEn), the interstitial nucleus of the posterior limb of the anterior commissure (IPAC) and the caudate-putamen (CPu; **Figure 2B**). In addition, six more injections were centered in the CxA, three of them extended to the Pir and the other three to the ACo. The pattern of labeling was similar in all four restricted injections (case 1324 is illustrated in **Figure 3**) and the labeling in the non-restricted injections was consistent with the pattern observed, that is, they showed the same retrograde labeling plus additional labeling that could be attributed either to the Pir or the ACo, depending on the case.

Since the size of the tracer injections had to be small to avoid affecting the adjoining areas, the retrogradely labeled cell bodies presented, in general, a few granules of DAB precipitate in the perinuclear cytoplasm, and the density of labeled cells was low along the brain. The highest density of labeled cells was present in the olfactory system. In addition, a moderate number of labeled somata was observed in other brain structures, such as the bed nucleus of the accessory olfactory tract (BAOT) and ACo in the amygdala, the nucleus of the horizontal limb of the diagonal band (HDB) and the magnocellular preoptic nucleus (MCPO) in the basal forebrain and the ventral tegmental area (VTA) in the midbrain.

#### Retrograde Labeling in the Olfactory System

**FIGURE 4:**
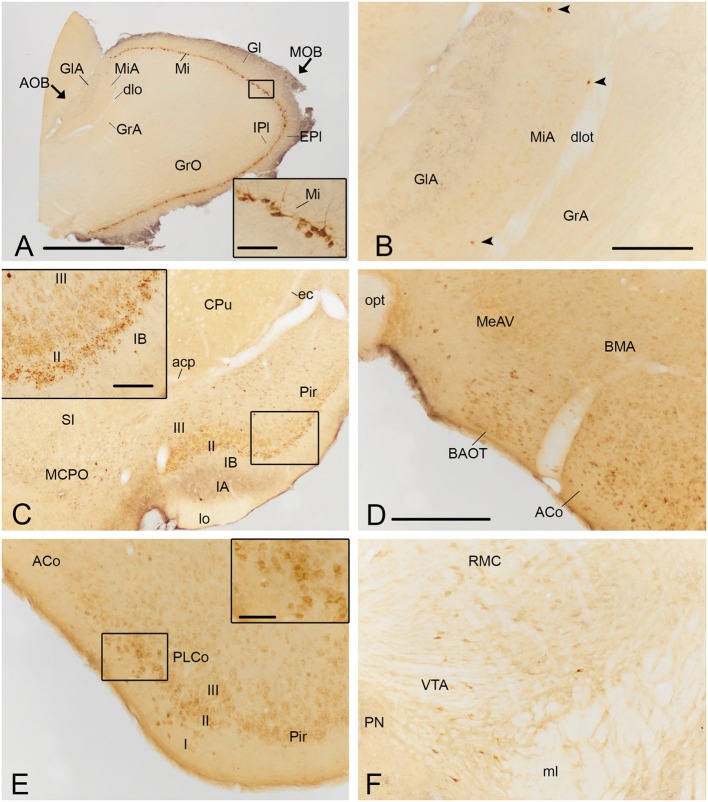
**Photomicrographs of frontal sections illustrating the retrograde labeling through the mouse brain of animals receiving a FG injection in the CxA**. The images correspond to the retrograde labeling present in cases 1324 **(A,B,D,E)**, 1331 **(C)** and 1311 **(F)**. **(A)** Retrograde labeling in the MOB. The inset in **(A)** depicts the darkly stained somata of mitral cells, which in some cases also show labeling in the proximal dendrites. **(B)** A few retrogradely labeled neurons in the mitral cell layer of the accessory olfactory bulb (arrowheads). **(C)** Labeling in the rostral part of the piriform cortex (Pir) and MCPO. The inset illustrates the labeled cells in layer II of the Pir, mainly located in its external part. An intensely labeled neuron is present in layer III (arrowhead). **(D)** Moderate amount of retrogradely labeled cells in the BAOT tract and ACo. **(E)** Retrogradely labeled neurons in the PLCo. The inset shows labeled cells mainly located in layers I and II of the PLCo. **(F)** Retrograde labeling in the VTA. For abbreviations, see list. Scale bar in **(A)**: 1 mm. Scale bar in **(B)**, valid for **(C)**: 500 μm. Scale bar in insets in **(A,C)**: 100 μm. Scale bar in **(D)**, valid for **(E,F)**: 250 μm. Scale bar in inset in **(E)**: 50 μm.

Injections of FG into the CxA gave rise to very dense labeling throughout the mitral cell layer of the MOB (**Table 1**; **Figure 3A**). The labeled somata were darkly stained and in some cases, the labeling extended into the proximal dendritic tree (**Figure 4A**). Few labeled cells were also observed in the external and internal plexiform layers of the MOB. In the injections of FG located in layer II of the CxA the mitral cell layer of the AOB (MiA) presented scarce labeling (**Table 1**, **Figures 3A** and **4B**), whereas in those injections located in layer I and extending into the *lo*, labeling in the MiA was denser (not illustrated).

Within the olfactory cortex, the Pir and the dorsal endopiriform nuclei (DEn) displayed the highest density of labeled cells. Labeling in the Pir showed a heterogeneous distribution, with a dense population of labeled cells in the anterior Pir but scarce labeling in its posterior division (**Table 1**, **Figures 3B–E**). In the anterior part of the Pir, labeled somata were mainly located in the outer part of the layer II and displayed a few granules of FG in each cell body (**Figure 4C**, inset). Some darkly stained somata were also present in the layer IA just below the *lo* and in layer III (**Figure 4C**). The DEn and VEn also showed heterogeneous labeling distribution, with their rostral parts displaying more labeled somata than their caudal ones (**Table 1**, **Figures 3B–E**). Finally, the dorsal *tenia tecta* (DTT) and the posterior part of the anterior olfactory nucleus showed only a few retrogradely labeled cells (**Table 1**).

#### Retrograde Labeling in the Amygdala

Within the amygdala, labeling was concentrated in the chemosensory nuclei, while the rest (deep) amygdaloid nuclei were almost devoid of labeled cells (but see below). Among the olfactory amygdala, a moderate density of labeled cells was observed in the ACo (**Table 1**), with more labeled cells located in its rostral than in its caudal aspect (**Figures 3C–E** and **4D,E**). Similarly, the PLCo showed a heterogeneous distribution of labeled neurons, which were mostly located in its rostral part (layers I and II; **Figures 3E** and **4E**), whereas the caudal PLCo was virtually devoid of labeling. In addition, the nucleus of the olfactory tract (LOT) also showed some scarce labeled somata mainly located in layer II (**Figure 3C**). Within the vomeronasal amygdala (**Table 1**), a moderate density of labeling was found in the BAOT (**Figures 3D** and **4D**), especially after tracer injections located superficially. Concerning the medial amygdaloid nucleus, scarce labeled cell bodies were restricted to its anterior (MeA) and posteroventral subnuclei (MePV) (**Table 1**, **Figures 3D,E** and **4D**), and the PMCo showed a very low density of labeled cell bodies (**Table 1**; not shown). Finally, the dorsal and ventral parts of the anterior amygdaloid area (AAD and AAV, respectively) showed scarce labeled cells (**Figure 3C**).

In the rest of the amygdaloid complex, only the medial division of the central amygdaloid nucleus (CeM) and the anterior part of the basomedial amygdaloid nucleus (BMA) showed some labeled cells (**Table 1**, **Figures 3D,E**).

#### Retrograde Labeling in the Cerebral Cortex

Within the cerebral cortex, only mesocortical areas (Palomero-Gallagher and Zilles, 2015) such as the lateral entorhinal cortex (LEnt) and the posterior part of the agranular insular cortex (AIP) displayed labeled somata (**Table 1**, **Figures 3B–D**), the density of which was always very low. In the two superficial injections, very scarce labeling was also observed in the ventral part of the agranular insular cortex (AIV; not shown).

#### Retrograde Labeling in the Septum and Basal Forebrain

Within the subcortical telencephalon, with the exception of the septohippocampal nucleus (SHi) in which very few labeled cells were observed (**Table 1**), only basal forebrain structures showed retrograde labeling. There, a moderate density of labeled cells was present in the HDB and MCPO (**Table 1**, **Figure 3B**). Labeled somata in the HDB were concentrated in its boundary with the olfactory tubercle (Tu) at rostral levels (not shown), and were more homogeneously distributed caudally where labeled cells also appear in the MCPO. In addition, the ventral aspect of the vertical diagonal band (VDB) showed scarce labeling (**Table 1**). Within the striato-pallidum, the ventral pallidum (VP) and semilunar nucleus (SL) showed scarce labeled cells (**Table 1**) and the *substantia innominata* (SI) and the IPAC presented very scarce labeling (**Table 1**, **Figures 3B,C** and **4C**).

### Retrograde Labeling in the Diencephalon, Midbrain and Brainstem

In our injections into the CxA no retrograde labeling was observed in the hypothalamus or thalamus, with the exception of a few labeled somata in the nucleus *reuniens* of the thalamus observed in one case (**Figure 3D**). Within the midbrain, the VTA showed a moderate number of labeled somata (**Table 1**, **Figures 3F** and **4F**). In addition, in the brainstem, the dorsal raphe and the rostral linear nucleus of the raphe showed scarce and very scarce labeling respectively (**Table 1**). The three injections that gave rise to a deposit of tracer along the micropipette track affecting the CPu showed labeled cells in the *substantia nigra*, which were present both in its *pars compacta* and *pars reticulata* (**Table 1**, **Figure 3F**). Remarkably, the injection without this leaking in the CPu did not show labeled cells in these structures.

#### Contralateral Labeling

Although the FG injections gave rise mainly to ipsilateral retrograde labeling, a few labeled cells (with very few granules of DAB precipitate) were also observed in the contralateral Pir and LOT.

#### Neurochemistry of Afferent Projections to the CxA

**FIGURE 5:**
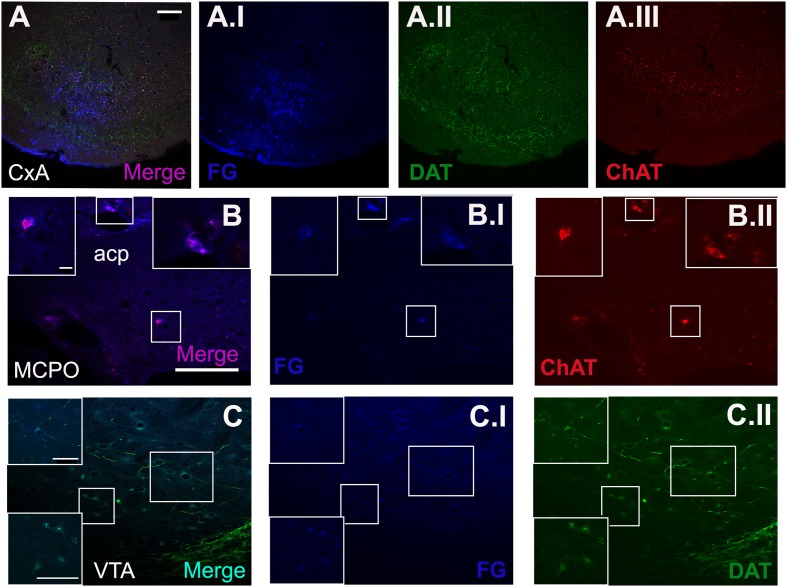
**Triple immunofluorescence staining for FG, choline acetyltransferase (ChAT) and dopamine transporter (DAT)**. **(A)** Merged picture of the expression of the three markers in the injection site. **(B)** Shows the double-positive cells for ChAT and FG in some cells of the MCPO. **(C)** Displays the co-localization of DAT and FG in the cell population of the VTA. The blue, green, and red channel of each photomicrograph is showed. All images were captured from the coronal section of animal 1324. Scale bar in **(A)**: 100 μm. Scale bar valid for **(B,C)**: 100 μm. Scale bar inset in **(B)** is valid for all the insets in **(B)**: 10 μm. Scale bar inset in **(C)** is valid for all the insets in **(C)**: 50 μm.

The triple immunofluorescence staining for FG, ChAT and DAT confirmed that the restricted injections were located in CxA (**Figure 5A**). Within the basal forebrain, we observed some cells double positive for ChAT and FG, located in the MCPO (**Figure 5B**). In addition, in the ventral midbrain a group of cells of the VTA showed co-localization of FG and DAT (**Figure 5C**).

### Anterograde Labeling after BDA Injections into the CxA

**FIGURE 6:**
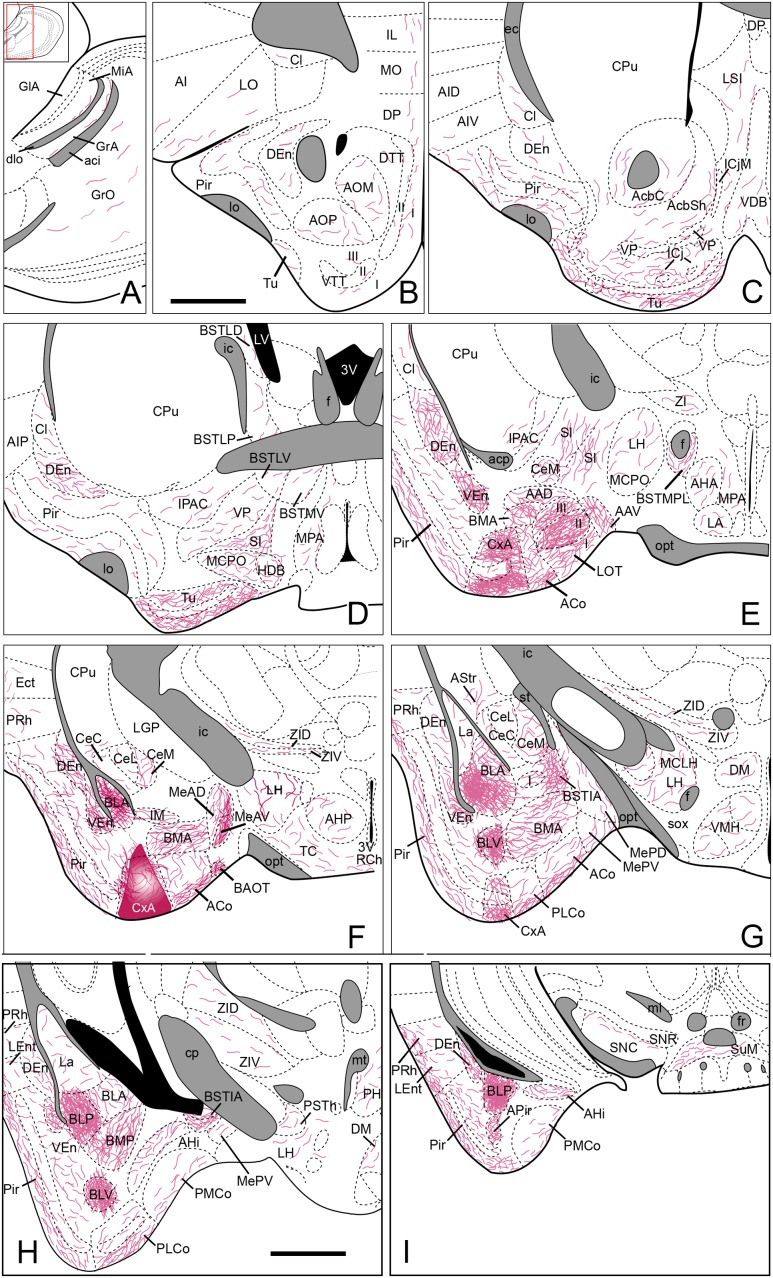
**Summary of the distribution of anterograde labeling following a BDA injection in the CxA, plotted onto semi-schematic drawings of parasagittal** **(A)** and frontal **(B–I)** sections through the mouse brain. The injection site is depicted in **(F)**. **(B)** is rostral, **(H)** is caudal. The semi-schematic drawings are based on the case 1331, which presented a restricted injection with no tracer deposit along the pipette track. For abbreviations, see list. Scale bar in **(B)**, valid for all panels: 1 mm.

Nine injections were located in the CxA, four of which were restricted to this nucleus (cases 1240, 1261, 1320, and 1331, **Figures 2D–H,J**). In these four cases the injections involved the superficial and deep layers of the CxA. The injection 1240 presented a small deposit of tracer along the micropipette track in the AAD and VEn (**Figures 2D–G**). Each of the restricted injections gave rise to a consistent pattern of anterograde labeling (case 1331 is shown in the **Figure 6**). In addition, five more injections were mainly located in the CxA, but involved other nuclei such as the Pir (*n* = 2), the AAD and VEn (*n* = 1) or the ACo (*n* = 1). These non-restricted injections showed a pattern of labeled fibers similar to that of the restricted injections although, as expected, additional fiber labeling was observed.

The efferent projections of the CxA reciprocate the inputs from the piriform cortex and the olfactory amygdala. In addition, the CxA projects densely to the basolateral amygdaloid nucleus and the olfactory tubercle. Extratelencephalic projections of the CxA are very scarce, and target mainly several hypothalamic structures.

#### Anterograde Labeling in the Olfactory System

**FIGURE 7:**
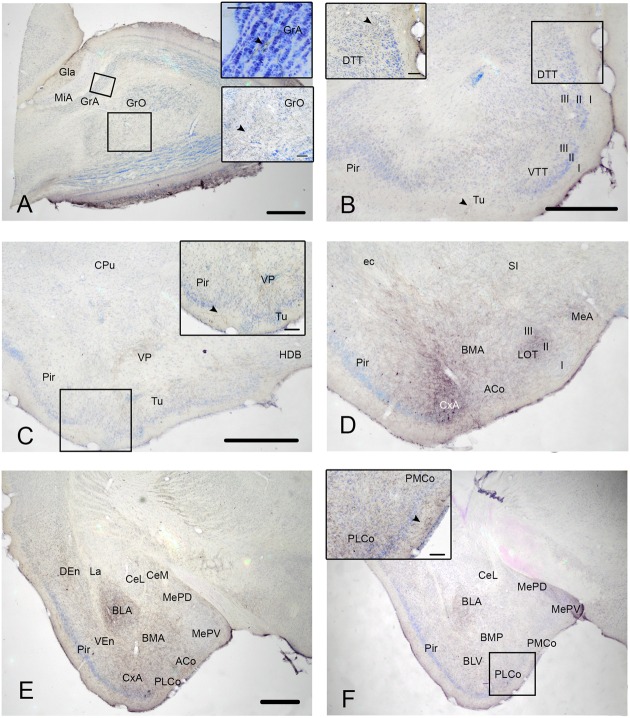
**Photomicrographs of frontal sections through the mouse telencephalon, illustrating the anterograde labeling resulting after BDA injections in the CxA**. **(A)** Anterograde labeling in the MOB and accessory olfactory bulb (AOB). Insets in **(A)** shows high magnification details of the labeled fibers in the granular layer of both bulbs. **(B)** Anterograde labeling present in the olfactory cortex and the basal forebrain. The inset shows the presence of labeled fibers in the DTT. **(C)** Moderate presence of labeled fibers in the basal forebrain and also in the olfactory cortex. Inset in **(C)** shows a high magnification detail of the labeled fibers in the piriform cortex (Pir) and in the olfactory tubercle (Tu). **(D)** Dense anterograde labeling next to the injection site, as well as in other amygdaloid nuclei. **(E,F)** Anterograde labeling at caudal level of the amygdaloid complex. The inset in F shows the difference number of labeled fibers between the PLCo and PMCo. For abbreviations, see list. The arrows point the presence of labeled fibers. Scale bar in **(A)** (valid for **E,F**): 0.5 mm. Scale bar in **(B)** (valid for **C,D**): 0.5 mm. Scale bars in insets: 0.1 mm.

The injections in the CxA gave rise to a scarce labeling in the granular layer of the MOB (**Table 1**, **Figure 7A**). In the AOB, tracer injections affecting all layers of the CxA (**Figures 2D–G** and **6F**) gave rise to a very small amount of anterogradely labeled fibers present mainly in the granular cell layer (**Figure 6A**). In addition, very scarce labeling was observed at the bottom of the MiA (**Figures 6A** and **7A**).

Regarding the rest of olfactory structures, the dorsal and ventral endopiriform nuclei and the caudal Pir showed the highest density of labeled fibers (**Figures 6B–I**). Anterograde labeling in the Pir showed a heterogeneous distribution of fibers, with a higher amount of labeled fibers in its caudal part (**Table 1**, **Figures 6C–I** and **7B–F**) located mainly in its layers I and III. It is noteworthy that labeled fibers were present in the whole layer I, and not restricted to the sublayer Ib. The tenia tecta presented scarce labeling in its ventral (VTT) and dorsal (DTT) divisions (**Table 1**, **Figures 6B** and **7B**). In addition, the posterior, lateral, medial and ventral parts of the anterior olfactory nucleus showed scarce labeling (**Table 1**, **Figure 6B**).

#### Anterograde Labeling in the Amygdala

The chemosensory amygdala showed a high density of anterogradely labeled fibers. Moreover, very dense anterograde labeling is also present in the basolateral (and, to a less extent, in the basomedial) amygdala (**Table 1**).

In the olfactory amygdala (**Table 1**), BDA injections gave rise to very dense anterograde labeling in the LOT (**Figures 6E** and **7D**) centered in its layer 2. In addition, the PLCo (**Figures 6G,H** and **7E,F**), the APir (**Figure 6I**) and ACo (**Figures 6E–G**, and **7D,E**) showed a dense presence of labeled fibers. The labeling in PLCo and ACo is mainly present in their external plexiform layers.

Regarding the vomeronasal amygdala (**Table 1**), injections in the CxA yielded a heterogeneous projection to the medial amygdala. The injections showed a very dense labeling in the MeA, centered in its anteroventral part (MeAV) (**Figures 6F** and **7D**), while in the posterodorsal and posteroventral subnuclei the labeling showed an heterogeneous distribution, being moderate in the anterior region and decreasing to very scarce caudally (**Figures 6G** and **7E**). In addition, a dense presence of labeled fibers was observed in the AAD, AAV and BAOT (**Figures 6E,F**). Finally, only a moderate labeling was observed in PMCo (**Figures 6H,I** and **7F**).

The basolateral complex displays a very dense fiber labeling in the anterior (BLA), posterior (BLP) and ventral part of the basolateral amygdaloid nucleus (**Figures 6G–I** and **7E,F**). In addition, the BMA (**Figures 6F,G** and **7D,E**) and the posterior part of the basomedial amygdaloid nucleus (**Figures 6H** and **7F**) presented a moderate level of stained fibers. On the other hand, the amygdalohippocampal area (AHi) displayed a scarce number of labeled fibers (**Figures 6H,I**). Lastly, the lateral amygdala (La) showed very scarce anterograde labeling from CxA (**Figures 6G,H** and **7E,F**).

In the central nucleus, moderate anterograde labeling was observed in the CeM (**Figures 6E–G** and **7E**), and very scarce labeling appeared in the capsular and lateral parts (**Figures 6F–G** and **7E**). In addition, the intercalated nuclei of the amygdala showed dense anterograde labeling, which was only moderate in the main part of the intercalated amygdaloid nucleus. Finally, the amygdalostriatal transition area (AStr) presented a low number of labeled fibers (**Figure 6G**).

#### Anterograde Labeling in the Bed Nucleus of the Stria Terminalis (BST)

The injections in the CxA resulted in very scarce anterograde labeling in the BST (see **Table 1**) with the exception of its intraamygdaloid division (BSTIA), which showed a dense level of labeled fibers (**Figures 6G,H**). A very low number of labeled fibers was observed in the dorsal, ventral and posterior parts of the lateral BST (**Figure 6D**), in the medial ventral and anteromedial BST divisions, as well as in the different subdivisions of the medial posterior BST.

#### Anterograde Labeling in the Cerebral Cortex

In the cerebral cortex, only the perirhinal cortex showed dense labeling (**Table 1**, **Figures 6F–I**). In addition, moderate anterograde labeling was present in the LEnt (**Figures 6H,I**). Very scarce levels of labeled fibers were also observed in the agranular insular cortex and different frontal cortical areas (infralimbic cortex, dorsal peduncular cortex, lateral and medial orbital cortices, **Figures 6B,C**) as well as in the claustrum (**Figures 6B–E**) and ectorhinal cortex (**Figure 6F**).

#### Anterograde Labeling in the Septum and Basal Forebrain

In the lateral septal complex, a very scarce number of fibers was present only in its intermediate part (**Figure 6C**) and in the septohypothalamic nucleus (see **Table 1**). In contrast, the diagonal band showed a moderate density of fiber labeling in the HDB/MCPO (**Figure 6D**), and scarce labeling in the VDB (**Figure 6C**).

Within the ventral striatum, moderate labeling appeared in the olfactory tubercle (**Figures 6C,D** and **7C**), with axons mainly surrounding the ICj. In addition, a small number of fibers were observed in the nucleus *accumbens* (**Figure 6C**). Within the striato-pallidal complex, moderate anterograde labeling was observed in the SI (see **Table 1**, **Figures 6D,E**). Scarce anterograde labeling was present in the SL and VP (**Figures 6C,D** and **7C**), and very scarce fiber labeling was observed in the IPAC (**Figures 6D,E**).

#### Anterograde Labeling in the Prethalamus and Thalamus

The injections in the CxA resulted in very scarce anterograde labeling in the thalamic complex (**Table 1**). In the prethalamus, fiber labeling could be observed only in the *zona incerta*, and in the thalamus anterograde labeling was present only in the anterior and posterior parts of the paraventricular thalamic nucleus (**Figures 6E–H**).

#### Anterograde Labeling in the Preoptic Area

Since the preoptic area is now considered to be part of the subpallium, instead of the hypothalamus (Puelles and Rubenstein, 2015), we describe labeling in this area in a separate section. A very scarce amount of anterograde labeling was present in the medial (**Figures 6D,E**) and lateral (not showed) preoptic areas.

#### Anterograde Labeling in the Hypothalamus

Following injections in the CxA, anterograde labeling appeared in many hypothalamic nuclei, although labeling was scarce all over the areas (**Table 1**).

At anterior levels, a low density of labeled fibers was observed in the anterior and posterior areas of the anterior hypothalamus (**Figures 6E,F**) as well as in the latero-anterior hypothalamic nucleus (**Figure 6E**).

In the tuberal region, a scarce presence of labeled fibers was observed in the lateral hypothalamic area (**Figures 6E–H**) and in the magnocellular nucleus of the lateral hypothalamus (**Figure 6G**). In addition, very scarce fiber labeling was observed in the retrochiasmatic area and the *tuber cinereum* (**Figure 6F**), the posterior hypothalamic area (**Figure 6H**), the dorsomedial (**Figures 6G,H**) and ventromedial hypothalamic nuclei (VMH) (**Figure 6G**) and in the parasubthalamic nucleus (**Figure 6H**).

Finally, in the mamillary region a scarce amount of fibers was observed in the supramamillary nucleus (**Figure 6I**). In addition, very scarce labeling was present in the medial and lateral mamillary nuclei, and in the dorsal and ventral premamillary nuclei (**Table 1**).

#### Anterograde Labeling in the Midbrain and Brainstem

The midbrain and brainstem presented a few labeled fibers only in the interpeduncular nucleus and in the medial aspect of the parabrachial nucleus (**Table 1**).

#### Contralateral Labeling

We have not consistently observed contralateral projections after the injections of BDA in CxA. In one case a solitary fiber could be observed in the contralateral BSTMA.

## Discussion

To our knowledge, this is the first study focused on the description of the afferent and efferent connections of the CxA. The data available in the literature correspond to studies mainly performed in rats describing the efferent projections of some structures that project to the Pir, in some of which projections to the CxA can be observed (see, for a review, Martínez-García et al., 2012; Olucha-Bordonau et al., 2015). One recent study in rats obtained restricted injections in the CxA (Pro-Sistiaga et al., 2007), but reported only the resulting labeling in the olfactory bulbs. In mice, our laboratory has previously reported the projections from the CxA to the ventral striatum, using both anterograde and retrograde tracing (Novejarque et al., 2011), as well as the connections with the vomeronasal amygdala (medial nucleus: Pardo-Bellver et al., 2012; Cadiz-Moretti et al., 2016; and posteromedial cortical nucleus: Gutierrez-Castellanos et al., 2014). It is important to note that most of the previous neuroanatomical works did not differentiate between the Pir and the CxA, and it is necessary to study their descriptions and illustrations to assign the observed labeling in the ventral part of the Pir (at the appropriate rostrocaudal levels) to what we considered now is the CxA according to the ChAT labeling (**Figure 1**) and the atlas of Paxinos and Franklin (2004).

**FIGURE 8:**
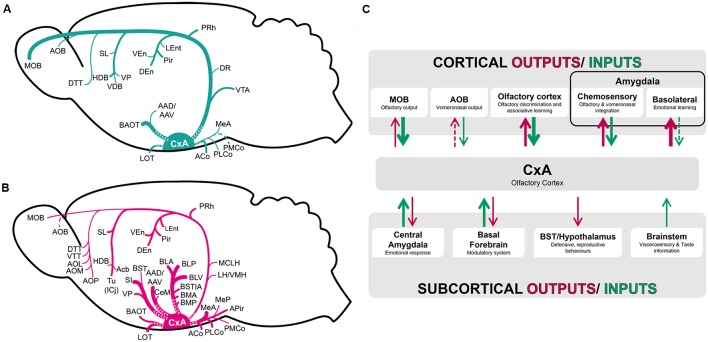
**Schematic representation of the pattern of afferent** **(A)** and efferent **(B)** projections of the CxA described in the present work. **(C)** Functional interpretation of cortical and subcortical projections to the CxA. The thickness of the arrows roughly represents the density of the projections.

Our tracing experiments show that CxA presents a relatively restricted set of afferent connections, as it receives mainly projections from the olfactory system and basal forebrain, with minor afferents from the amygdala and the VTA. The efferents of the CxA are also somewhat limited, but include important projections to the chemosensory and associative (BLA and BMA) amygdala, to the ventral striatum, and minor projections to the BST complex, cortical structures including the entorhinal and perirhinal cortices, and thalamic and hypothalamic structures (**Figure 8**).

From the methodological point of view, it is worth noting that restricted injections in the CxA had to be reduced in size, to avoid affecting the neighboring Pir or ACo. The small size of these injections may have resulted in relatively reduced anterograde and retrograde labeling, and therefore we may slightly underestimate the density of the projections to and from some of the nuclei.

### Connections With the Olfactory System

Our results confirm that the CxA receives convergent projections arising from the main and accessory olfactory bulbs (**Figure 8A**), corroborating previous works done with anterograde and retrograde tracers (Pro-Sistiaga et al., 2007; Kang et al., 2009; Cádiz-Moretti et al., 2013). The MOB is the main sensory input to the CxA, sending much stronger projections than the AOB, which sends only minor projections. The afferents from the MOB and AOB are originated from the entire mitral cell layer, apparently lacking a topographic organization. These results are consistent with previous reports in rats and mice (in rats: Mohedano-Moriano et al., 2007; Pro-Sistiaga et al., 2007; in mice: Kang et al., 2009). In contrast to this organization of the projection to the CxA, the projection from the MOB to the medial amygdala apparently arises mainly from its ventral aspect (Kang et al., 2009). The mitral cells of the ventral MOB projecting to the Me are activated by opposite sex odors but not by same sex odors or predator odors (Kang et al., 2009). This suggests that only a fraction of MOB output cells conveys information to the ‘vomeronasal’ amygdala. These results raise the question of which type of olfactory and vomeronasal information reaches the CxA. According to the well-known role of the amygdala in processing emotional information (Martínez-García et al., 2012), it would be expected that projections arising from the olfactory bulbs to this structure send chemosensory information related to conspecifics or predators, or maybe to learned odors associated with aversive experiences (such as in olfactory fear conditioning) or appetitive experiences (such as sexual encounters). However, to our knowledge no functional data are available to test this hypothesis.

Differences in density of retrograde labeling were observed in the AOB depending on which layers were affected by the injections in the CxA. The superficial injections (mainly centered in layer I) displayed more labeling in the AOB than injections centered in layer II. The AOB fibers that project to CxA mainly innervate layer I, with only some fibers coursing inward to innervate deep layers (in rat, Pro-Sistiaga et al., 2007; in mouse, Cádiz-Moretti et al., 2013). The fibers in layer I were described as thick and varicose, suggesting that they may form *en passant* synapses in the distal portions of the dendrites of the CxA neurons (Pro-Sistiaga et al., 2007; Cádiz-Moretti et al., 2013). Therefore, the differences in the observed retrograde labeling in the AOB could be explained by the uptake of the tracer by these fibers in the superficial injections, and consequently we consider unlikely the possibility of the uptake of the tracer by fibers damaged in the *lo* by the micropipette tip.

Our findings showed that the CxA receives substantial projections arising from the Pir (**Figure 8A**), a hodological feature shared with the Me and PMCo. Previous studies have also shown that the olfactory cortex sends projections to the CxA (with the CxA corresponding to the ventral aspect of the Pir at the level of the ACo (Luskin and Price, 1983; McDonald, 1998; Martínez-García et al., 2012), in agreement with our results. Following CxA injections, the retrogradely labeled somata were mainly located in the layer IIa. This layer is mainly composed by neurons with three or less apical dendrites and a short (or inexistent) basal dendrite (Haberly and Price, 1978a). Since the apical dendrites of these neurons are located in layer I, their input is probably dominated by the axons originated by the MOB. This is consistent with the view of the CxA as an area devoted to the processing of olfactory information.

In addition, the CxA receives more projections from the rostral (or prepiriform area, Haberly and Price, 1978a,b) than from the caudal Pir, whereas it projects more strongly to the caudal Pir (innervating mainly layers I and III). In agreement with this observation, the posterior Pir presents more bidirectional connections with the amygdala than the anterior Pir (Haberly, 2001; Majak et al., 2004). Therefore, the connectivity of the CxA suggests that it is a transition area between the Pir and the amygdala, and it does not clearly belong to either the layer Ib system or the layer II system as defined by Haberly and Price (1978a,b). Electrophysiological data has revealed that, while the anterior Pir processes sensory information of the odorants, the posterior Pir shows associative encoding characteristics (Calu et al., 2007). Thus, the anterior Pir may send information related to the features of the odorant to the CxA, while the CxA may relay to the posterior Pir olfactory information processed to include the behavioral significance of the odorant, provided by the cholinergic and dopaminergic inputs as well as by inputs from other amygdaloid structures.

Although the connections of the CxA with the Pir and LEnt cortices, as well as with the olfactory amygdala (LOT, ACo and PLCo, see below), resemble those of the Pir, there are also important differences. In addition to the minor input from the AOB, the CxA differs from the rostral Pir mainly in the lack of inputs from the anterior olfactory nucleus as well as in the lack of contralateral projections to the olfactory cortex (Haberly and Price, 1978a; Luskin and Price, 1983; Martínez-García et al., 2012). In spite of these differences, it is clear that the CxA is strongly related with the rest of the olfactory system.

### Connections With the Amygdaloid Complex and BST

The CxA receives minor projections from the olfactory amygdala, with the ACo sending moderate projections and the PLCo and LOT scarce projections (**Figure 8A**). In agreement with our findings, Santiago and Shammah-Lagnado (2004), illustrated fibers in the CxA (see their Figure 2J) following their injections in LOT. Regarding to the vomeronasal amygdala, the CxA received light projections from the PMCo, Me and AAD/AAV, and moderate ones from the BAOT (**Figure 8A**). Similar results were obtained with anterograde injections in the PMCo (Canteras et al., 1992; Gutierrez-Castellanos et al., 2014) and Me (Canteras et al., 1995; Pardo-Bellver et al., 2012), with minor differences regarding to the density of the labeling observed. These differences may be explained in part by the small size of the CxA injections in the present work, as well as different criteria to classify the labeling as moderate or scarce.

In summary, the CxA shows light afferences from most of the olfactory and vomeronasal structures. The reciprocal projections are notably denser (**Figure 8B**), especially those targeting the olfactory amygdala. This suggests that the CxA receives elementary olfactory information directly from the olfactory bulbs and from the anterior Pir, and relays it to the chemosensory amygdala for further processing.

Regarding the deep amygdaloid nuclei, the CxA receives only very scarce projections from these structures (**Figure 8A**). In contrast, the projection from the CxA to the basolateral and basomedial nuclei are very important (**Figure 8B**), suggesting that it influences the emotional information (defined as information related to challenges for the survival of organisms, including, at a minimum, responses involved in defense, fluid and energy homeostasis, and reproduction, see LeDoux, 2012) processed in these structures (Martínez-García et al., 2012). Therefore, by means of the projection from the CxA to the associative amygdala, odor information may be linked to information about unconditioned stimuli reaching the basolateral and basomedial amygdaloid nuclei, and thus mediate olfactory learning (**Figure 8C**).

Concerning to the BST complex, it does not give rise to projections to the CxA, and receives only minor projections from it (**Figures 8B,C**), indicating a small influence on visceromotor information processing in these structures.

### Connections With the Cerebral Cortex

Our results showed that cortical structures (leaving apart the olfactory cortex) send virtually no projections to the CxA (**Table 1**). The CxA gives rise to minor projections to the mesocortical (prefrontal, insular and ectorhinal) areas (Palomero-Gallagher and Zilles, 2015) but projects densely to the PRh. Again, by this pathway olfactory information reaches a multimodal associative area.

The CxA shows also minor projections to the claustrum and moderate reciprocal connections with the DEn. Recent studies have shown that these two structures are developmentally related (Puelles, 2014; Watson and Puelles, 2016) and form a histogenetical unit derived from the embryonic lateral pallium (expressing the transcription factor Nr4α2) different from that originating the VEn (the ventral pallium). The Pir and amygdala are also derivatives of the ventral pallium. The functional meaning of the interconnection with the claustro-endopiriform domain is currently unclear, but on the basis of these developmental considerations it seems more related to information processing in the cerebral cortex than in the olfactory system.

Our results also show that the CxA has minor reciprocal projections with the LEnt (**Table 1**, **Figures 8A,B**). The LEnt has been considered part of the olfactory cortex (Luskin and Price, 1983), since it receives direct projections from the MOB. The connections with the CxA are probably part of the connectivity with other structures of the olfactory system. The LEnt has recently been shown to modulate the activity in the Pir and, from a functional point of view, to be involved in fine odor discrimination (Chapuis and Wilson, 2013). The projections to the CxA may be similarly involved in modulating the activity in the olfactory amygdala.

### Connections With the Septum and Basal Forebrain

The results presented in this work, together with hodological data available in the literature, indicate that the basal forebrain gives rise to a common input to the CxA and other cortical amygdaloid nuclei. Previous works have reported basal forebrain projections to the Me (Cadiz-Moretti et al., 2016), the LOT (Grove, 1988) and PMCo (Gutierrez-Castellanos et al., 2014) similar to those described in this work. The projections arising from the diagonal band were previously reported with HRP injections in the Pir (Haberly and Price, 1978b).

It is widely accepted that the AChE-positive nuclei of the amygdala present this reactivity because of their afferent connections from cholinergic neurons in the basal forebrain, which are mainly located in the medial septum, VDB, HDB, VP, MCPO and SI (Semba, 2000). In the present work we have shown that this is the case also for the CxA. The studies related to the possible role of the cholinergic system of the basal forebrain have been mainly focused on the basolateral amygdala (within the amygdaloid complex) and on the Pir (within the paleocortical structures). The basolateral nucleus and the Pir receive their cholinergic innervation from the medial septum, VDB, HDB, VP, MCPO and SI (Nagai et al., 1982; Woolf et al., 1984; Heckers et al., 1994). Woolf et al. (1984) suggested that the cholinergic system is mainly implicated in modulating the responses of other inputs. The cholinergic afferents received by the basolateral amygdala in rats are involved in modulating learning and memory processes related to emotional paradigms (that is, involving the use of unconditioned stimuli with either aversive or appetitive value, McIntyre et al., 1998; Vazdarjanova and McGaugh, 1999). In the case of the Pir, the cholinergic input modulates odor discrimination learning by means of its synapses with intra-cortical association fibers (Chapuis and Wilson, 2013). Given the transitional nature of the CxA, its cholinergic inputs may be modulating olfactory learning and memory processes.

### Projections to the Preoptic Area, Hypothalamus and Thalamic Complex

Our results showed that the preoptic area, hypothalamus and thalamic complex do not provide afferent connections to the CxA, and receive only very light projections from this structure (**Figure 8B**). The thalamic outputs are centered in the paraventricular nucleus, while the projections to the preoptic area and hypothalamus lightly innervate several structures at preoptic, anterior, tuberal and mamillary levels (see **Table 1**). These efferent projections are not restricted to areas involved in reproductive, defensive or aggressive behaviors (Simerly, 2015). Therefore, these minor projections may allow the olfactory information processed in the CxA to have a slight influence in the variety of behavioral outputs mediated by the preoptic area and hypothalamus.

### Connections With the Midbrain and Brainstem

The VTA in the midbrain and the raphe in the brainstem showed minor projections to the CxA (**Figure 8A**), while the CxA projects (very lightly) to the interpeduncular nucleus and medial parabrachial nucleus (MPB).

As our double-labeling experiments have shown, the VTA contains the cells of origin of the dopaminergic innervation of the CxA (Martínez-García et al., 2012). Since dopamine in the mesolimbic pathway has been implicated in signaling the incentive salience of reward-related stimuli (Berridge and Robinson, 1998), it is tempting to suggest that the dopaminergic input of the VTA may be implicated in processing the incentive salience of olfactory stimuli. In addition, since biologically relevant olfactory stimuli probably induce olfactory learning, this dopaminergic projection may contribute to the plasticity underlying this learning process.

The CxA gives rise to a small projection to the MPB, which is considered to be a gustatory relay center, implicated in processing taste information and in the learning of taste aversion (Yamamoto et al., 1994). Therefore, the CxA projection may send to the MPB information related to incoming olfactory information.

### Possible Functional Roles of the CxA

The CxA shares the majority of its afferent connections with the adjacent Pir, with both of them being more connected with neighboring olfactory structures and the basal forebrain and to a minor extent with the amygdala (**Figure 8C**). On the other hand, CxA differs from the adjacent ventromedial Pir in its stronger cholinergic and dopaminergic innervation (present results; Martínez-García et al., 2012), as well as in its light vomeronasal input and in the lack of afferents from the anterior olfactory nucleus and in the lack of commissural connections with the contralateral olfactory cortex. These features suggest that CxA is indeed a transition area between the adjacent Pir and the cortical amygdala, which may be specialized in processing information of particularly arousing odors, since both the cholinergic and dopaminergic input are likely related to attentional processes (Sarter and Bruno, 2000) and salience (Berridge and Robinson, 1998) properties, respectively. It is also possible that cholinergic and dopaminergic signaling in the CxA play a role in plasticity related to olfactory learning in this structure, although to our knowledge there is currently no experimental evidence supporting this hypothesis.

## Author Contributions

BC-M, EL, and FM-G designed research; BC-M and MA-A performed experiments. BC-M, MA-A, and CP-B analyzed data; BC-M, MA-A, CP-B, and EL wrote the paper. EL and FM-G revised the final version.

## Conflict of Interest Statement

The authors declare that the research was conducted in the absence of any commercial or financial relationships that could be construed as a potential conflict of interest.

## Abbreviations

I: layer 1
II: layer 2
III: layer 3
3V: 3rd ventricle
4V: 4th ventricle
AAD: anterior amygdaloid area, dorsal part
AAV: anterior amygdaloid area, ventral part
AcbC: nucleus accumbens, core
AcbSh: nucleus accumbens, shell
ACo: anterior cortical amygdaloid nucleus
aci: anterior commissure, intrabulbar part
acp: anterior commissure, posterior part
AHA: anterior hypothalamic area, anterior part
AHi: amygdalohippocampal area
AHP: anterior hypothalamic area, posterior part
AI: agranular insular cortex
AID: agranular insular cortex, dorsal part
AIP: agranular insular cortex, posterior part
AIV: agranular insular cortex, ventral part
AOB: accessory olfactory bulb
AOP: anterior olfactory nucleus, posterior part
APir: amygdalopiriform transition area
Astr: amygdalostriatal transition area
BAOT: bed nucleus of the accessory olfactory tract
BLA: basolateral amygdaloid nucleus, anterior part
BLP: basolateral amygdaloid nucleus, posterior part
BLV: basolateral amygdaloid nucleus, ventral part
BMA: basomedial amygdaloid nucleus, anterior part
BMP: basomedial amygdaloid nucleus, posterior part
BST: bed nucleus of the *stria terminalis*
BSTIA: BST, intraamygdaloid division
BSTLP: BST, lateral division, posterior part
BSTLV: BST, lateral division, ventral part
BSTMA: BST, medial division, anterior part
BSTMV: BST, medial division, ventral part
CeC: central amygdaloid nucleus, capsular part
CeL: central amygdaloid nucleus, lateral division
CeM: central amygdaloid nucleus, medial division
Cl: claustrum
CPu: caudate putamen
CxA: cortex-amygdala transition zone
DAB: 3,3^′^-diaminobenzidine
DEn: dorsal endopiriform nucleus
dlo: dorsal lateral olfactory tract
DP: dorsal peduncular cortex
DR: dorsal raphe nucleus
DTT: dorsal *tenia tecta*
ec: external capsule
Ect: ectorhinal cortex
EPl: external plexiform layer of the main olfactory bulb
f: fornix
FG: Fluorogold
GrA: granule cell layer of the AOB
GrO: granular cell layer of the main olfactory bulb
HDB: nucleus of the horizontal limb of the diagonal band
I: intercalated nuclei of the amygdala
ic: internal capsule
ICj: islands of Calleja
IL: infralimbic cortex
IM: intercalated amygdaloid nucleus, main part
IPAC: interstitial nucleus of the posterior limb of the anterior commissure
IPl: internal plexiform layer of the main olfactory bulb
LA: lateroanterior hypothalamic nucleus
La: lateral amygdaloid nucleus
LEnt: lateral entorhinal cortex
LH: lateral hypothalamic area
LPO: lateral preoptic area
LO: lateral orbital cortex
lo: lateral olfactory tract
LOT: nucleus of the lateral olfactory tract
LSI: lateral septal nucleus, intermediate part
LV: lateral ventricle
MCPO: magnocellular preoptic nucleus
Me: medial amygdaloid nucleus
MeA: medial amygdaloid nucleus, anterior subnucleus
MeAD: medial amygdaloid nucleus, anterodorsal part
MeAV: medial amygdaloid nucleus, anteroventral part
MePD: medial amygdaloid nucleus, posterodorsal subnucleus
MePV: medial amygdaloid nucleus, posteroventral subnucleus
Mi: mitral cell layer of the main olfactory bulb
MiA: mitral cell layer of the AOB
ml: medial *lemniscus*
MO: medial orbital cortex
MOB: main olfactory bulb
MPA: medial preoptic area
opt: optic tract
PH: posterior hypothalamic area
Pir: piriform cortex
PLCo: posterolateral cortical amygdaloid nucleus
PMCo: posteromedial cortical amygdaloid nucleus
PN: paranigral nucleus
PRh: perirhinal cortex
PSTh: parasubthalamic nucleus
PV: paraventricular thalamic nucleus
RCh: retrochiasmatic area
RLi: rostral linear nucleus of the raphe
RMC: red nucleus, magnocellular part
SHi: septohippocampal nucleus
SHy: septohypothalamic nucleus
SI: *substantia innominata*
SL: semilunar nucleus
st: *stria terminalis*
SuM: supramamillary nucleus
Tu: olfactory tubercle
VDB: nucleus of the vertical limb of the diagonal band
VEn: ventral endopiriform nucleus
VMH: ventromedial hypothalamic nucleus
VP: ventral pallidum
VTA: ventral tegmental area
VTT: ventral tenia tecta
ZI: *zona incerta*
